# Prominent Thebesian Veins in Association With Takotsubo Cardiomyopathy

**DOI:** 10.7759/cureus.16204

**Published:** 2021-07-06

**Authors:** Priyadarshini Dixit, Ruchi Shah, Yashwant Agrawal, Ruchit Shah, Anjani Rao

**Affiliations:** 1 Internal Medicine, Saint Joseph Mercy Oakland, Pontiac, USA; 2 Interventional Cardiology, Cedars Heart Clinic, Chandler, USA; 3 Cardiology, Saint Joseph Mercy Oakland, Pontiac, USA

**Keywords:** takotsubo cardiomyopathy, cardiology, thebesian veins, cardiac catheterization, apical ballooning

## Abstract

Thebesian veins are microvascular connections from the coronary arterial supply directly into the heart chambers. While they play an important role in providing nourishment to the myocardium by maintaining adequate perfusion, they are also responsible for a physiologic right to left shunt in the body’s circulation. We present a case report of this rare anatomic finding of extensive Thebesian veins causing acute coronary syndrome and Takostubo cardiomyopathy.

## Introduction

Thebesian veins are a small group of non-valvular veins that drain blood from the walls of the cardiac chambers directly into their respective cavity [1,2]. These veins are considered to be a normal anatomical variation of little pathological significance. However, there are case reports which described the association between the presence of prominent Thebesian veins and myocardial ischemia.

Literature review shows how enlarged Thebesian veins can cause atypical chest pain, ischemic electrocardiographic changes as well as troponin elevation. The proposed mechanism is likely coronary stealing phenomenon in cases of large Thebesian veins causing coronary ischemia and subsequent acute coronary syndrome (ACS) symptoms. Myocardial perfusion is reduced secondary to above and hence can lead to features similar to Takatsubo cardiomyopathy which were seen in our patient including the apical ballooning with impaired ejection fraction. 

EKG findings are typical for T wave inversions in at least one of the precordial leads and associated ST changes [3-6]. Troponin elevation may be seen in some cases but not all. Left heart catheterization performed in the majority of cases showed prominent Thebesian veins draining into the left ventricle. These cases, in combination with the case discussed below, provide evidence that prominent enlarged Thebesian veins can cause hemodynamically significant changes leading to ACS signs and symptoms including peculiar EKG changes as mentioned above.

## Case presentation

A 67-year-old Caucasian female presented to the Emergency Department with complaints of left-sided chest pain associated with lightheadedness. Her past medical history was significant for cerebro-vascular accident s/p right carotid endarterectomy, hypertension, hyperlipidemia, and chronic obstructive pulmonary disease.

She complained of ongoing intermittent episodes since the past three weeks of dull, 6/10 in severity, non-radiating pain over the left side of the chest which was aggravated with inspiration and relieved by aspirin. During this presentation, she also complained of lightheadedness. The initial examination including vital signs was unremarkable.

**Figure 1:**
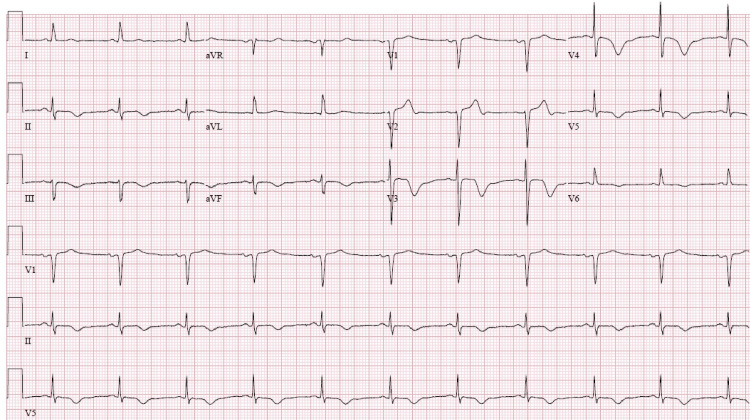
EKG at presentation demonstrating T wave inversion in leads II, III, aVF and V3-V6

**Figure 2:**
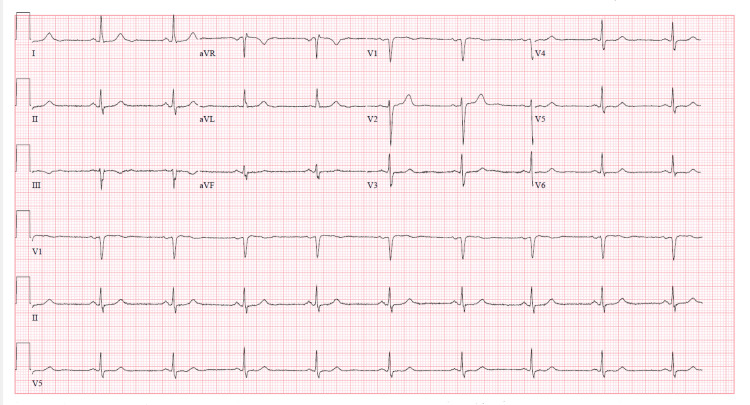
EKG one year ago showing no T wave inversions

**Figure 3:**
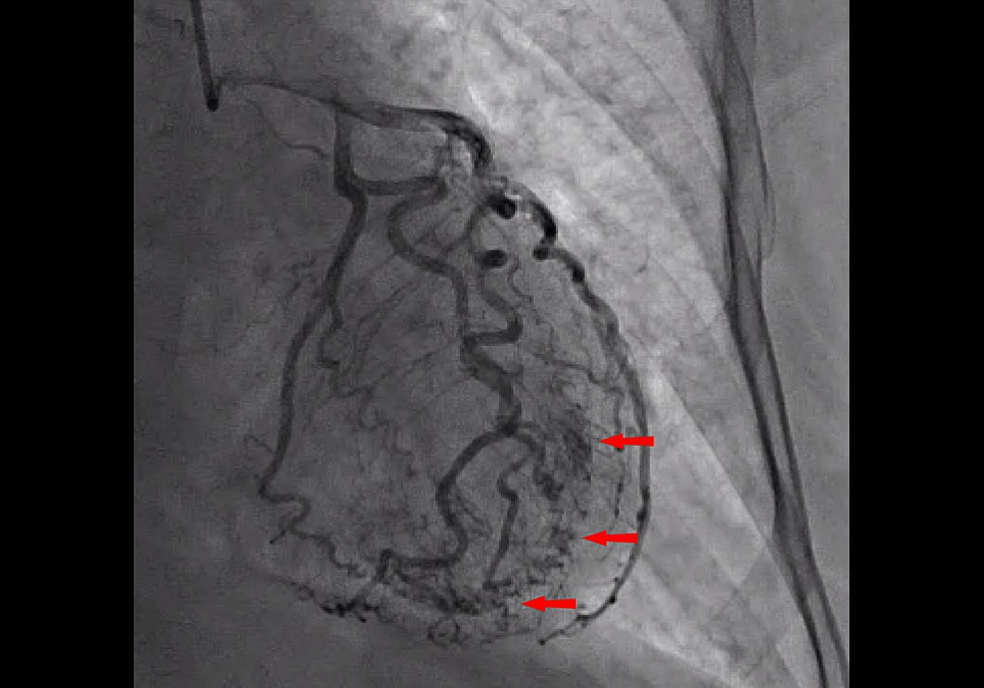
Picture taken from left main angiography showing patent coronary arteries and prominent Thebesian veins as demonstrated by the red arrows

**Figure 4:**
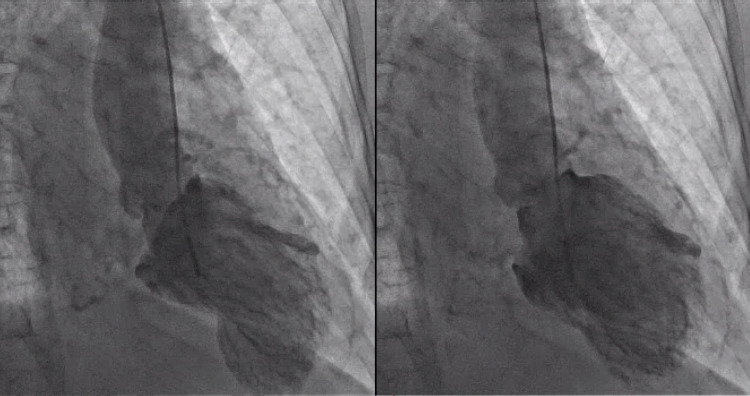
Left ventriculogram showing apical ballooning consistent with Takotsubo cardiomyopathy

EKG revealed T wave inversion in leads V3 through V6 (Figure 1). These changes were new compared to the previous EKG done one year prior (Figure 2). Serum troponin level was elevated with initial Troponin I of 0.82 ng/ml (normal < 0.03 ng/ml). Conventional heparin drip was initiated depending on partial thromboplastin time (PTT) to maintain PTT goal 60-100. She underwent left heart catheterization with bilateral coronary angiogram which demonstrated patent coronary arteries and prominent micro-fistulae forming communications (Figure 3). The left ventriculogram demonstrated prominent Thebesian veins draining into the left ventricle and 40% ejection fraction with apical ballooning consistent with the diagnosis of Takotsubo cardiomyopathy (Figure 4).

Though unable to quantify, it was obvious that the venous blood flow through the Thebesian veins was more than normal since her ejection fraction was impaired.

With these findings on cardiac catheterization with minimal coronary artery disease (CAD) but prominent Thebesian veins with impaired ejection fraction, the patient’s chest pain symptoms were likely secondary to coronary steal phenomenon due to prominent Thebesian veins.

The patient was treated with low-dose aspirin, clopidogrel, beta-blocker, and nitrates as anti-anginal drugs. No intervention was performed due to Thebesian veins being small in caliber. Improvement in symptoms was noted with initiation and optimization of her CAD medications and the patient was advised for lifestyle modification to minimize her CAD risk.

## Discussion

The venous drainage system of the heart is largely divided into the greater cardiac venous system which runs on the epicardial surface providing up to 70% drainage and the lesser cardiac venous system which drains the myocardial layer, comprising the remaining 30%. Forming the lesser cardiac venous system are the Thebesian veins, a small group of nonvalvular veins embedded in the cardiac walls which help to drain blood from the myocardium directly into their respective cavity [7,8]. Thebesian veins are more important during the embryonic development of the heart and contribute to a lesser extent to adult heart circulation. They are more abundant in the right atrium as compared to all the heart chambers [9]. Thebesian veins are essential in providing an alternate route of perfusion, nourishment, and drainage to the myocardium. On the other hand, since it is associated with the direct emptying of blood into the cardiac chambers, it also contributes to the physiologic right to left shunting of the body’s circulation.

By ensuring adequate perfusion to the myocardium, Thebesian veins play an important role in transporting oxygen as well as nutrients to the heart tissue which has the highest percentage of oxygen extraction amongst body tissues as well as transports carbon dioxide and other waste material back into the circulation.

There are two known physiologic right to left shunts in the adult cardiovascular circulation: bronchial veins supplying the conducting airway of lungs and Thebesian veins of the left side of the heart draining deoxygenated blood into the oxygenated blood containing left atrium and ventricle.

When enlarged or present in increased numbers, Thebesian veins can cause left to left shunting, meaning that it draws downstream blood from the arterial system again into the heart chamber creating an alternative pathway for circulation and decreasing the effective cardiac output. This mechanism causes a loop-like pathway diverting blood from the aorta to the coronary arteries back into the left ventricle and then again into the aorta [10]. Such alternative circulatory pathways can lead to diastolic volume overload and decreased cardiac output leading to exercise limitations and dyspnea. Essentially, it is a form of coronary stealing phenomena causing anginal symptoms due to myocardial ischemia.

Though echocardiogram with Doppler flow and cardiac MRI can be useful for diagnosis of Thebesian veins, coronary angiography is needed for definitive diagnosis. Unfortunately, treatment options are still limited from a surgical standpoint including coronary bypass grafting or transmyocardial laser revascularization (TMLR) for poor surgical candidates. TMLR creates channels in the left ventricular wall to improve blood flow between the ventricular lumen and myocardial muscle, in turn, improving the oxygen supply to the myocardium. From a medical standpoint, use of beta-blockers and possibly nitrates are currently the center of treatment in addition to modifying other risk factors for CAD as well as other contributing comorbidities.

## Conclusions

This case report provides more evidence of the presence of Thebesian veins as a cause of ACS secondary to coronary steal phenomenon causing myocardial ischemia and also exacerbating the left to left shunting. Takotsubo syndrome in this case might be caused by one of the pathophysiologic mechanisms explained above. However, further studies need to be conducted to confirm the clinical relevance of this particular association.
